# What role do young people believe Universal Basic Income can play in supporting their mental health?

**DOI:** 10.1080/13676261.2023.2256236

**Published:** 2023-09-08

**Authors:** Elliott A. Johnson, Hannah Webster, James Morrison, Riley Thorold, Alice Mathers, Daniel Nettle, Kate E. Pickett, Matthew T. Johnson

**Affiliations:** a Social Work, Education and Community Wellbeing, Northumbria University, Newcastle upon Tyne, UK; b Turn2Us, London, UK; c Universitat Autònoma de Barcelona, Barcelona, Spain; d The RSA, London, UK; e Independent Researcher; f Ecole Normale Supérieure-PSL, CNRS, Paris, France; g Department of Health Sciences, Faculty of Science, University of York, York, UK

**Keywords:** Adolescent mental health, Universal Basic Income, anxiety and depression, social determinants of health, public engagement

## Abstract

The proportion of 16- to 24-year-olds in England reporting a longstanding mental health condition increased almost 10-fold between 1995 and 2014. Studies demonstrate an association between income and anxiety and depression, with bi-directional effects. There is also emerging evidence that cash transfers may mitigate, prevent or delay those conditions. This article presents qualitative data exploring the relationship between income and anxiety and depression and the prospective impact of Universal Basic Income (UBI) as a public health measure. Data was gathered from citizen engagement workshops with 28 young people aged 14–22 from Bradford, England. We present four findings: (i) participants believe that the current work and welfare system has a detrimental impact on their mental health; (ii) most participants believe that UBI would have positive impacts on their mental health by virtue of reducing financial strain; (iii) most participants appear to favour a UBI scheme with larger payments than have traditionally been proposed; (iv) participants believe that there are non-financial benefits of UBI, such as reduction in stigma.

## Introduction

The crisis in mental health among young people is an epidemic with worrying and far-reaching implications. Between 2009 and 2020, around one in three British 16- to 24-year-olds met self-reported clinical threshold scores for anxiety and depression (Parra-Mujica et al. 2023). Those levels were reached in the wake of the Global Financial Crisis, austerity and Brexit, but before the COVID-19 pandemic, the Russian invasion of Ukraine and the cost-of-living crisis. Each of these events has reduced the UK’s economic, social and health resilience, with national institutions and services facing unprecedented pressure and an increasing number of those in work now also in poverty (JRF 2022). There is an expanding body of evidence that the increase in anxiety and depression among young people and the decline in socioeconomic conditions are related, especially since the adoption of austerity measures in 2010 (Bell and Blanchflower 2019). In that context, cash transfers have been proposed and implemented in several countries to improve mental health outcomes among young people (see Zimmerman et al. 2021 for a systematic review of impact of cash transfers on children and young people in low-income and middle-income countries). This is essential, as the Health Foundation’s *Young People’s Future Health Inquiry* highlights that the ‘effects of social stressors on young people may not be apparent early in life but becomes biologically embedded in the first few decades of life (Jordan, Kane, and Bibby 2019).

This article reports the findings of citizen engagement workshops held in December 2021 with 28 young people (aged 12–24) in Bradford, UK aimed at exploring their understanding from a lived experience perspective the social determinants of mental health and the role of Universal Basic Income (UBI) – a largely unconditional, regular payment to all permanent residents to support people’s basic needs – as a public health measure to address anxiety and depression. This complements the quantitative evidence produced elsewhere through analysis of longitudinal datasets and trials of cash transfers from a range of countries (Johnson et al. 2020).

As detailed in the section X below, there is ample empirical evidence for a positive association between income and mental health of young people, both in the site of our study, England (Pitchforth et al. 2019) and other countries such as Finland (Haula and Vaalavuo 2022) and Australia (Landstedt, Coffey, and Nygren 2016). In response to these findings, other researchers have sought to explore the causal mechanisms between the association, such as the achievement of occupational aspirations (Gjerustad and von Soest 2012) or, among adults, employment status and the ratio of debt-to-assets (Zimmerman and Katon 2005). This paper takes a different approach to the problem. We adopt a participatory, qualitative method to explore young people’s perception and lived experiences of financial security and its relationship with mental. Additionally, we elicit the views of young people on policies such as UBI that could ameliorate the negative impact on mental health that low income produces. This paper’s focus on young people’s lived experiences of economic and welfare systems offers a novel contribution to the literature on the relationship between income and mental health. It does so via a participatory approach that aimed to reverse researcher-participant knowledge dynamics aimed at enabling young people to share their views as experts on their lived experience and to make more confident claims about the impact of policy.

We find that young people have a deep and nuanced understanding of the ways that external factors influence their mental health, suggesting that their views should be taken into account when developing policies. In particular, young people were enthusiastic about the prospect of UBI and believed that it would lead to better mental health outcomes for them and their peers. Participants identified several mechanisms through which this may happen such as a changing relationship to work, greater financial security and greater equality. Participants also expressed views that ran counter to the expectation that they would support UBI out of their own financial self-interest. Among those who completed the post survey workshop, just four of 22 expressed support for the most financially generous scheme presented, with almost two-thirds (14/22) voting for the middle option. These empirical findings contribute to an increased understanding of young people’s views of both existing and prospective policies.

In the paper, we first present a brief history of UBI, followed by a review of some of the evidence on the relationship between income and mental health. We then present our method and its justification. This is followed by the results and discussion, in which we examine three key issues. First, we explore young people’s evaluation of the current work-welfare system, its fairness, its capacity to advance their interests, and its impact on their mental health. We then explore perceptions of the prospective impact of UBI on their mental health. We do this via examination of both quantity and security of income and reframing of welfare in relation to cultural factors, such as stigma, as well as psychosocial goods, such as agency, relationship formation and planning. We finally consider the role that conditional supplements may play in supporting those with differing or additional needs.

## What is UBI and why is it relevant?

Typically, UBI is defined as a universal and unconditional cash payment from the government to every member of a society. The intellectual history of UBI can be traced back to radical thinkers, both liberal and socialist, towards the start of the modern period, such as Thomas Paine, John Stuart Mill and Thomas More (Ghatak and Maniquet 2019), though the similarity of such ideas to contemporary UBI proposals are debated (Jäger and Vargas 2023). Interest in UBI can be traced back to the 1960s, spreading from the US to Europe, with the creation of the Basic Income Earth Network in 1986 (Jäger and Vargas 2023).

On the left, UBI is supported for its ability to create conditions for real freedom (van Parijs 1995) or more narrowly by freeing workers from labour insecurity (Standing 2012). On the right, UBI is seen as an elegant way of removing state bureaucracy and wage regulations (Friedman 2020).

The most relevant literature for this study pertains to the impact of UBI on health and particularly mental health. A systematic scoping review by Gibson, Hearty, and Craig (2020) found mixed effects on health, with no effect on some outcomes, but strong effects in others, such as birth weight and mental health. For example, a longitudinal study of payment made to all community members of a Cherokee reservation reported positive effects on adult and child mental health across multiple waves (Costello et al. 2010).

Johnson et al. (2022) have proposed three pathways through which UBI can positively affect health: resource scarcity, impact on chronic stress, and behaviour. Together, these offer the framework through which we approached the study. Given the link between economic insecurity and increased mental health problems and rising insecurity in the UK among young people (Ofsted 2022), we explored whether UBI could provide an effective mitigation method, based on previous empirical and Johnson et al.’s (2020; 2022) theoretical framing. UBI may also be an especially effective policy for young people because the main UK benefit types are usually only available to those aged 18+ (Social Security Advisory Committee 2018, 21). Under 18s may be affected by the welfare system either through their families being in receipt of a benefit, in particular child benefit, or through representations of benefits in the media. UBI would affect everyone, and Reed et al.’s (2023) microsimulation modelling of the economic impacts of three UBI schemes provides evidence that UBI could lead to the kinds of effects that young people identify as important, from increasing income to reducing the inequality that participants feel in their everyday interactions. The relationship between income and mental health.

Between 1995 and 2014, the proportion of 16-to 24-year-olds in England reporting a longstanding mental health condition increased almost 10-fold (Pitchforth et al. 2019). Reported rates of self-harm (5.3% to 13.7%) and attempted suicide (1.3% to 2.2%) also increased from 2000 to 2014 among 16–24s (Clarke, Pote, and Sorgenfrei 2020, 7). The consequence is a generation of young people affected by potentially avoidable forms of mental health problems, while healthcare and public services become stretched to the point of breaking. In England alone, there were 420,314 open referrals to child and adolescent mental health services (CAMHS) in February 2022 (NHS Digital 2022), a 54% increase since the same month in 2020 (NHS Digital 2020). There is, it appears, no sign of the crisis abating.

While policy has often understandably focused on improving coping strategies and increasing the efficiency of reactive healthcare services (Garratt, Laing, and Long 2022), interest is growing in addressing the social drivers of anxiety and depression at source (House of Commons Health and Social Care Committee 2021, 11–13) as part of the prevention agenda espoused by recent governments (DHSC 2018). A developing body of evidence indicates that those conditions are strongly affected by social determinants (Lessof et al. 2016) such as income, wealth, education, social capital and opportunity.

While some GPs have called for cash prescriptions (Johnson, Degerman & Geyer 2019), a range of organisations, health bodies, community groups and politicians have called for trials of Universal Basic Income (UBI). Some of the authors of this study (Johnson et al. 2022) have presented a theoretical UBI model of impact which suggests that schemes that provide regular, uninterrupted access to cash support have the capacity to improve outcomes by reducing poverty, inequality-related stress and health diminishing behaviour. Indeed, where relevant outcomes are measured, studies of cash transfer schemes around the world indicate significant improvements in mental health and wellbeing (Gibson, Hearty, and Craig 2020).

While causality between income and mental health may be bidirectional (Wilson and Finch 2022), Parra-Mujica et al.’s (2023) analysis of the Understanding Society UK Household Longitudinal Study indicates not only a correlation between income and anxiety and depression among young people, but that income is the primary driver of the latter. Young people aged 16–24 in the lowest household income quintile were 5.8 percentage points more likely on average to report an SF-12 Mental Component Summary (MCS) score of ≤45.6, indicating clinically significant symptoms of depressive disorder, than their peers in the highest quintile (Parra-Mujica et al. 2023). Increases in income in that age group were associated with an improved MCS score. These results support the results from Villadsen et al.’s (2023) analysis of the UK Millenium Cohort Study, which finds that young people from the lowest two income quintiles had an increased likelihood of experiencing psychological distress than higher quintiles. Microsimulation modelling of the economic impact of UBI schemes at three payment levels has indicated that their introduction would lead to substantial increases, on average, to the incomes of those in all but the highest income groups, while slashing rates of poverty and inequality (Reed et al. 2023). The pandemic and cost-of-living crisis, which has left more than two in five households in fuel poverty (Bradshaw and Keung 2022), has given added impetus to research on the possible role of basic income as a mental health intervention.

Other researchers have proposed several different causal mechanisms. In research based on 49 life story interviews in Finland, Rikala (2020) suggests that agency may play a role in the mental health problems of economically marginalised young people. Institutional policies curtail depressed young people’s agency and inhibit their potential paths out of distress. In Norway longitudinal survey research indicates that an association between an inability to realise occupational aspirations and an increase in anxiety levels (Gjerustad and von Soest 2012).

Given this growing quantitative evidence of a relationship between income and mental health in young people, and UBI’s potential ability to deal with the issue, there is a need to understand young people’s own perceptions and preferences through qualitative work.

## Methods

### Data collection

We designed and ran online participatory engagement workshops in December 2021 with 28 young people aged 14–24 from Bradford, a Northern English city with high levels of deprivation. Participants were recruited via the Born in Bradford (Wright, McEachan, and Mathai 2022) and ActEarly (Wright et al. 2019) research programmes. Born in Bradford is a cohort study programme based in the city and ActEarly is an associated programme aimed at improving the health of children in the city. Their excellent connections and reputation in Bradford, as well as shared aims, made them ideal recruitment partners.

Two workshops were held for each of four age groups (14–16; 16–18; 18–21; 21–24). Online workshops were chosen in order to mitigate risks resulting from the COVID-19 pandemic as well as the flexibility and accessibility it offered participants (Flanagan et al. 2015). Access to suitable facilities for participants without personal internet access (Flanagan et al. 2015) and alternative accessible options were offered though not requested. We used an informed consent and screening survey, which ran from 4 to 24 November 2021, to solicit initial socio-demographic data on age, ethnicity, religion, long-term conditions and disability, education and employment, receipt of benefits (personally and in the household), income, and accommodation type. This enabled a targeted allocation of places within oversubscribed workshops to ensure they included a diversity of participants with protected characteristics, particularly disabled people, due to their constituting an excluded group specifically affected by welfare reforms.

In advance of the workshops, those who were selected to take part were invited to complete a pre-workshop survey, which ran from 23 November to 6 December 2021 and asked about self-rated health, perceived stress, anxiety and depression symptoms as well as diagnosis, self-perception, subjective socioeconomic status, social comparisons, concerns about transitioning from education, and caring responsibilities. 22 out of 28 workshop participants completed this survey.

Areas for discussion in the workshops were identified from previous scoping work the project team had undertaken (e.g. Johnson et al. 2022) as well as entries from the pre-workshop survey. These areas were size of payment, needs-based supplements, distribution of payments for under 18s, and anticipated impacts of payments. The workshops and the co-production-based facilitation were designed to support participants’ ability to share sensitive information about their finances and mental health while also enabling discussion and development of ideas (Punch 2002). The sessions were run by the RSA using an adapted form of their Citizen Engagement Participatory Action Research with young people method (see Tejani and Breeze 2021).

The first set of workshops was designed to enable participants to think reflectively about the role of money in their lives and how it facilitates and/or inhibits their wellbeing. We also canvassed participants’ views on the current welfare system. The session closed with a brief introduction to UBI to understand young people’s first responses to the policy. The second session directly addressed young people’s perceptions of advantages and disadvantages of a UBI in turn. Participants were asked to imagine and share how a UBI could impact their own lives and mental health, and what they thought the wider societal impacts would be. We introduced three different UBI schemes and discussed their relative merits, how they should be paid for and whether under-18s should receive a payment directly or through a parent/guardian. We concluded the session by running an online poll to find out which scheme the participants preferred and whether and how they thought money should be distributed to under18s.

Finally, participants completed a post-workshop questionnaire, which was open for completion between 3 and 20 December 2021 and examined perception of the three UBI schemes and the impacts of their favoured scheme on their lives and health conditions. It also provided a debrief. 22 our of 28 workshop participants completed this survey.

Participants were remunerated with shopping vouchers up to the value of £100 if they completed the surveys and workshops to address issues of exploitation in research and to encourage diverse recruitment (Largent and Fernandez Lynch 2017). Partial completion was remunerated on a *pro rata* basis.

The protocol and research materials are available at https://doi.org/10.17605/OSF.IO/968FT.

### Participant characteristics

50 participants took part in the consent and screener survey, with 28 each taking part in the pre-workshop survey and workshop, and 22 taking part in the post-workshop survey. Some workshop participants did not take part in the pre-workshop survey and vice versa.

Women and girls were overrepresented throughout, at 80% in the screener and 82% in the workshops. The sample was otherwise relatively diverse, which reflects Bradford’s population of which 25.5% were ethnically Pakistani and 56.7% White British in the 2021 Census (ONS 2022) (Table 1).

**Table 1.** Workshop participant characteristics.

Gender	
Female	23
Male	5
Workshop age group	
14–16	5
16–18	11
18–21	6
21–24	6
Ethnicity	
White British	15
Non White British	13
Religion	
No religion	13
Any religion	15
Employment or education	
Full-time education	23
Other	5
Respondent or chief earner receiving benefits (other than Child Benefit)	7
Disability status (according to Equality Act definition)	
Non-disabled	21
Other	7
Diagnosed anxiety or depression	
Not Diagnosed	18
Other	10
Family wealth relative to friends	
Poorer	8
About the same	9
Richer	1
Don’t know	4
No response	6
Managing financially (16+)	
Finding it very difficult	1
Finding it quite difficult	1
Just about getting by	5
Doing alright	7
Living comfortably	4
Under 16	2
No response	6
Household income	
Under £24,000	8
£24,000–£35,999	5
£43,000–£74,999	5
£75,000 or more	6
Don’t know	2
No response	2

### Analysis

Alongside survey data, we captured notes, recordings and transcripts from all eight workshops and, using the digital whiteboard programme Miro, transferred all points made by participants. We then conducted thematic analysis (Braun and Clarke 2006), grouping these in the following categories which reflected our prompted questions: young people’s financial situation; relationship between money and mental health; perceptions of the current system; positives and negatives of UBI; how to pay for a UBI; level of UBI payment; UBI for young people (under-18s). Given the interconnected nature of the findings, in the final analysis below we group the four categories on UBI into two sections. To aid readability, we have proofread and condensed quotes where necessary. We used the participant surveys to contextualise analysis.

## Results

### Young people’s financial situation

Participants described the existing system of work, education and welfare as not working for young people. It was clear that across different income levels, participants felt pressure to keep up and were challenged to get by. Participants expressed a pervasive sense of financial insecurity necessitating difficult trade-offs.

Working takes up a lot of time and energy … [it] does affect your uni work, and if you’re juggling work and uni it limits time with your friends and family and quality time as you’re always anxious about workload. (Male, 21–24)

This spanned conversations about housing, welfare and wider economic systems and in some cases was related to experiences of insecurity earlier in life. Typically, older cohorts (18+) were more actively preoccupied with their financial situations given their reliance on a larger and steadier source of income than those aged under 18 who had financial support from their parents and considerably lower outgoings. One participant (male, 21–24) said that he was unable to sustain his initial approach at university of using spreadsheets for finances when he became too ‘overloaded’ and would then make a ‘really irrational purchase’, feeling that he could not ‘live this strategically all the time’. A participant (female, 18–21) said that she could not afford to go out with friends at the weekend and that being at home every day ‘makes you feel quite down’.

A trade-off was perceived by some participants as a decision about whether to focus on the present or the future. Some participants also shared how they opted to prioritise their future careers via study rather than ensuring financial wellbeing in the short term by seeking work. One participant (female, 21–24) age group said that ‘[studying] can mean you sacrifice things at the time and feel anxious about money but hopefully in the long run its worth it to get a degree’. There was also an awareness that the stresses of overwork, combined with the pressures of education, could have a detrimental effect on young people’s mental health.

On the other hand, one participant (female 21–24) said that in order to maintain positive mental health it was sometimes better to turn down paid work over an immediate improvement in their financial situation. Not all participants were in a position to do this, with some unable to avoid working long hours alongside their studies. Among those at university, a majority had jobs to cover the financial shortfall. This included working zero-hours contracts and others working multiple jobs to get by. One participant (female, 21–24) was undertaking around 10–20 h of online tutoring while on holiday.

### Relationship between money and mental health

Almost all participants in our workshops described having experienced anxiety and/or low mood, frequently linking these to their financial situation. Some participants had experienced or been diagnosed with prolonged mental health problems, with anxiety one of the most common issues mentioned. Participants described themselves as being surrounded by worries about money.

Some also described anxieties about the future, linked to their growing independence and other changes in their lives. Transition points, like moving into further education, were seen by participants as prominent drivers of potential anxiety on the basis that doing something ‘wrong’ could affect them into the future. For some participants, these feelings were compounded by work pressures, not having enough time to see friends and family and the pressure associated with comparing themselves to others with more money.

It sort of makes you feel like crap, especially when everyone around you has so much money in comparison to you … [It’s] really embarrassing to explain to people that you can’t do things … And then they look down at you. (Male, 21–24)

While anxiety was the emotion most commonly raised by participants in relation to their financial situation, some also reported feelings associated with depression. They often linked this with having to make sacrifices in order to pursue their interests, such as not seeing their friends and family or stopping their hobbies. Many participants described feelings of exhaustion, lethargy or being drained. Many also explored feelings of guilt when spending. The feeling was most pronounced among people who obtained money from their parents, with many observing that they felt different about spending money from different sources. For some participants from poorer families, this guilt meant that they avoided asking for money from family when possible.

Sometimes when I ask for money from my parents, I kind of feel guilty anyway, because I’m taking away money that they need for essentials and to help us have a roof over our heads. (Female, 14–16)

For, particularly older, participants who rely on parents for money, perhaps due to limitations on earning during study, there were reports that this can undermine independence, maturity and self-esteem. One participant (male, 21–24) described it as having an ‘emotional toll’ and as like being in ‘limbo’.

A range of factors was identified by participants in relation to their emotional state and mental health. The most common of these were described as stress or pressure which were linked to various aspects of young people’s lives. Participants were clear that the ‘mental load’ impact of financial distress on ‘cognitive bandwidth’ was significant and detrimental.

Being unemployed and only having money from student loans makes you need to plan all the time on how you spend your money … you can never relax, it’s just like anxiety and stress. (Female, 21–24)

It was also apparent that participants understood that financial insecurity involves layers of increasing complexity and seriousness that has the potential disproportionately to affect those from lower socioeconomic status backgrounds. As a participant (male, 21–24) noted ‘I think the main emotion is just stress and anxiety’, with debt, however, small, having a large and negative impact due to money problems in his childhood.

For participants in higher education, it was clear that those without parental financial support were likely to face increased pressures on their capacity to succeed in their studies. This compounded another driver of mental wellbeing issues relating to the inability to get a job due to a lack of experience, which in turn prevented them from gaining relevant experience, and concerns about finding work in the future. A participant (female, 18–21) noted that ‘I’m always worried that if, if I don’t do well now, I’ll [not] be able to make money in the future. So that’s like my main stress at the moment’.

The issues to do with subjective social status and social norms around ‘oney and status were felt by some participants to be amplified by erroneous media and social media representations of young people’s incomes and lifestyles, with one participant (female, 16–18) mentioning the representation of ‘super sweet 16s’ as well as the pressure of seeing peers with more money. There were further issues raised by a number of participants to do with a lack of confidence in their understanding about money issues such as tax and how education could improve the situation. The young people we spoke to were clearly very aware of the socioeconomic drivers of their stress, but unable to address them without substantial difficulty. For some, achieving balance in their lives is simply not possible in their present circumstances.

### Perceptions of the current benefits system

Participants often felt the delivery of the current welfare system was unfair, though responses were more mixed when it came to its underpinning design. This spanned concerns about payment thresholds and those who just miss out, the waiting period before any payment, and a one-size-fits-all approach to people’s needs which demonstrated ‘a lack of humanity’. One participant (female, 21–24) who had been in receipt of Universal Credit described having it stopped at the last minute through no fault of her own and how that compounded issues relating to housing and food security, including going ‘weeks without food’. She further expressed her anxiety and stress at the thought of ever going ‘back to being in such a bad place again’. One participant (female, 21–24) described the difficulty of the current needs-assessed system for disabled people through the lens of their partner’s experience of having not been awarded disability support but also struggling to undertake daily tasks. She further detailed the psychological impact on both of them of applying for Universal Credit:

we didn’t want to admit that we needed the help and support and we didn’t want to live on benefits. And then the whole process of applying for it was really complicated and confusing as well … We were very depressed for a long time. (Female, 21–24)

A number of participants reflected on the stereotypes about benefit claimants passed down from older generations and felt the stigma was a negative part of the current system, including the use of language relating to being a ‘burden on society’. We found that these perceptions of the current system most commonly influenced participants’ discussion of a UBI when it came to the level of payment.

A few participants considered the system fair and some considered it overly generous. Reflecting common media narratives, some perceived that people were ‘taking advantage of the system’ or scamming the government, citing family members who felt that the system was ‘too easy’. One participant (female, 14–16) feared that this might lead to slower administration and a resulting negative impact on ‘people who actually have those conditions’. In some instances, however, participants made a point of differentiating their own views from common narratives, with one participant (female, 21–24) group describing the news as having a tendency to ‘demonise people on benefits as … just lazy’.

Participants also had a nuanced awareness of the role that the benefits system plays in wider cycles of debt, poverty and inequality. One participant (male, 21–24) referenced the lengthy system and its being a ‘last port of call’ for people as creating a ‘cycle of … poverty, where they can’t seem to get out of it, because … help wasn’t there when they actually needed it’. Participants showed an intuitive understanding of the efficacy of preventative interventions, stating that people get into greater and greater financial trouble due to receiving insufficient upstream support. They also said that this led to a lower degree of social mobility and stress caused by a lack of financial security. The groups acknowledged differences among other young people, such as those who had higher levels of responsibility earlier in life.

### The potential impact of a UBI on social determinants of health

Having examined the current system in the first set of workshops, in the second set we explored the potential of UBI. Participants discussed the impact a UBI might have on individual relationships with work and the respective collective benefits or challenges this might bring. While there were concerns about universal benefits being channelled to those already financially comfortable, when a UBI was discussed alongside ideas for more progressive taxation there was support for the idea from almost all participants, with one participant (male, 21–24) stating that ‘All of us would be more happy, not just one of us. All a tiny bit happier everyday … There is [an] amplifier effect if everyone is happy’.

Of the 22 participants who took part in the post-workshop survey, 20 believed that they would be a little or a lot less stressed under their preferred UBI scheme and two felt it would have no impact on their stress. 17 felt they would be a little or a lot less anxious and three neither more nor less anxious. Two felt they would be a little or a lot more anxious. Finally, 18 believed that their mood would be a little or much better and four neither better nor worse.

19 participants took part in both the pre- and post-workshop surveys. In terms of whether their families are richer, poorer or about the same as those of their friends, 16 felt they would be about the same under UBI compared with seven currently. Of the 17 participants aged 16+, six imagined they would be living comfortably under their preferred UBI scheme and 11 that they would be doing alright. That compares with just two who were living comfortably in the pre-workshop survey, eight who said they were doing alright, five just about getting by, and one each finding it quite or very difficult.

### Increased security, better work-life balance and improved relationships

In the immediate term, participants welcomed the additional financial support that a UBI would provide, thereby alleviating some of the stress associated with paying bills. Several participants believed that a UBI would make them more comfortable and able to save, while others felt that it would reduce the pressure on them when thinking about the future.

There was discussion of how UBI might enable young people to work less, leading to improved mental health. With this additional free time, the participants imagined that they would have more time to relax and focus on their own wellbeing and personal relationships.

It would impact my life immensely- I would have time to do other things within my life such as extra curricular activities like music and sport and I would have more confidence to make my own business when I am older such as using the money to invest in a camera. (Female, 16–18)

One participant (male, 18–21) felt that with a UBI ‘all the stress of moving and having to find a job instantly would go away’. Many participants also suggested that a UBI would allow them to invest more time and energy in important relationships and could potentially ease interpersonal tensions caused by financial difficulties or financial dependence. One participant (male, 21–24) group felt that UBI could strengthen his relationship with his parents as their occasional financial reliance on him put him off spending time with them. On the other hand, a participant (female, 14–16) felt that a UBI could free her from the guilt she feels due to financial reliance on her parents, with an added benefit of helping her to ‘be more responsible and learn how to look after my own money’. One participant (female, 16–18) in the survey said that a UBI would mean her mother would not have to ‘overwork herself to make ends meet’ and could turn down extra shifts ‘instead of feeling pressure to do extra hours to avoid having anxiety over having unexpected bills’.

Participants recognised the potential of UBI to enable people to take on jobs that they really enjoy, rather than being pressured into settling for jobs simply to pay bills. They also reflected on how a UBI might change their plans for the future, either changing the field they aspired to be part of or allowing them to consider a more entrepreneurial path.

Ultimately, I would be able to take more risks as a UBI would provide a safety net which may lead to an accelerated career path and a quicker journey to financial stability, which in that position I would start to be able to pay back more into the system as my taxes would increase. (Post-workshop survey, male, 18–21)

Many participants praised the universality of the system, viewing UBI as an effective way of removing some of the barriers they had identified in the current system, such as thresholds and stigma. When paired with a progressive taxation to fund it, a UBI was also viewed as an effective means of reducing inequality as ‘everyone is equal and has a fair share’. Participants saw a UBI as an opportunity to ensure that ‘people started life on a more equal footing’ and expressed a hope that the policy would lead to a more meritocratic society. One participant (female, 21–24) with experience of being in receipt of benefits felt that a UBI would mean that ‘there’s not going to be that much judgement around you getting money from the government, because everybody is’. This is part of a wider belief that a reduction in inequality through UBI could result in reduced status anxiety and social comparison.

Participants also anticipated a positive economic impact beyond the payment itself as a result of increased productivity coming from improvements in wellbeing. Three cohorts referred directly or indirectly to the concept of the marginal propensity to consume by anticipating that a UBI would produce positive economic effects through redistribution to poorer people who are more likely to spend money and less likely to save it. In the post-workshop survey, one participant noted the likely cost savings on self:

I guess if people have more money, then that would have a knock-on effect for their health [and] the NHS wouldn’t be as strained as it is … I feel like there’s a lot of like ripple effects that would happen. (Female, 18–21)

An additional benefit to working fewer hours was that participants could focus more on studying. Some participants predicted that a UBI would result in improvement in their grades, while others viewed the educational benefits as being more expansive. One said that the additional time would allow them to pursue more extracurricular activities to gain a well-rounded education. Another felt that a UBI would allow young people to focus on courses that truly interest them, rather than those expected to deliver high financial returns in the future.

The notion of education as an investment was raised on several occasions. A UBI was seen as changing the mindset from young people trying to invest in themselves to a society investing in its young people’s futures collectively. Participants were clear about the need for ambition, aspiration and hard work. However, they were also clear that the existing system undermines their interests. In contrast, UBI was perceived as a common-sense response to a very straightforward problem: a lack of economic security. In the post-workshop survey, one participant summed up the challenges and opportunities that the modern economy is exposing:

Inequality is rising in the UK and reducing the gap should be priority for society … Computers are replacing jobs so, like, do we want to use this as an opportunity for equality, not increasing the gap between rich and poor? (Male, 21–24)

### Unfairness, irresponsibility and productivity

Typically, when a group was prompted to consider disadvantages of UBI, they first responded by saying that it would be unfair to make cash transfers to the richest in society. This was framed as some groups – variably ‘rich friends’, ‘people with generational wealth’ or ‘the middle class’ – getting something they do not need. Some concerns were alleviated when a UBI was paired with progressive taxation. However, there remained some apprehension that a UBI did not constitute a spending priority for the government and that the funding might be better used to improve healthcare provision. For example, one participant (female, 16–18) felt that the ‘upper class’ already have enough money and a UBI would therefore not be directed to people that truly need it. Ethical concerns were raised about what a UBI should be used for and whether there were societal risks related to the positives discussed earlier in conversations. Echoing some of the narratives about benefits claimants identified earlier, some participants felt that ‘people would take advantage’ of a UBI. This was expressed as concern that it may discourage personal financial responsibility, with participants voicing worries that people may spend it on materialistic things or ‘blow’ the money.

There was a fear that providing increased financial security could lead to potential productivity losses or job shortages among occupations perceived as being less desirable, whether due to the nature of the work or the pay. This was seen as the flip side to the personal benefits of taking on less or better work.

### Different UBI schemes

In discussions on payment levels, participants brought considered and reasoned perspectives into the pros and cons of higher and lower levels. Scheme 1 is based on the approximate amount paid to universal credit recipients. Scheme 3 is based on the average amount needed for recipients to meet the JRF’s (2021) minimum income standard in 2021, which is calculated to be the amount required a decent standard of living in the UK. Scheme 2 falls halfway between schemes 1 and 3 (Table 2).

**Table 2.** Amount received by different types of recipients under the draft UBI schemes.

Recipient type	Scheme 1	Scheme 2	Scheme 3
Under 18s	£40 per week	£40 per week	£40 per week
Single working age (18–64) person	£60 per week	£145 per week	£229.81 per week
Lone parent with two under 18s	£140 per week	£225 per week	£410.74 per week
Couple with two under 18s	£200 per week	£370 per week	£511.39 per week

#### Scheme 1

The starter scheme appealed to participants who had concerns about how recipients may spend a UBI. Some voiced their concerns that a higher payment may lead to it being spent on ‘reckless’ or ‘useless’ things. It was seen as appropriate that a UBI gives some additional help but ‘shouldn’t be to pay the rent’. Others said that taxpayers may not be in favour of a higher UBI. Opposition to this level of payment tended to relate to arguments that £60 per week is too low in most cases. It was felt by some that a UBI of £60 per week would not be much to fall back on especially for those on lower incomes.

#### Scheme 2

Scheme 2 proved popular with participants, though the majority of those in support of it opted for it as a middle ground. This perspective tended to emerge after a discussion of higher and lower levels. For some it was seen as a good baseline from which to begin experimenting. One participant saw it as the best scheme as it supports the current benefits system while ‘giving a decent amount of money’ and others added to the view that in combination with other benefits it was a fair level.

#### Scheme 3

Scheme 3 attracted a lot of praise for its commitment to ensuring a decent standard of living for all. Participants recognised that people need money to meet their essential needs and imagined this scheme as having a greater impact on those on low incomes, large families, disabled people and young people with no family support. It was also noted that the Minimum Income Standard does not cover the costs of luxuries, making it an appropriate means of state support. One participant from a less well-off background said that this payment could really help their family and others on low incomes. Downsides discussed mirrored wider concerns about a UBI, for example whether a payment, particularly at a higher level, could disincentivise work. One participant also expressed concern that the proposed expenditure might be more effectively spent on the NHS.

Of the 22 workshop participants who completed the post-workshop survey, 14 favoured Scheme 2. Four each favoured schemes 1 and 3 respectively.

### The practicalities of a transitional UBI

We also asked participants about their views on a UBI of £40 per week specifically for young people aged 14–17. Participants saw the benefits of a UBI for this age cohort in two broad categories: independence and improved relationships with friends and family. Participants saw it as an opportunity to provide a sense of independence for all young people, while also preparing them for the future. This was seen as having the greatest potential for young people who might be in difficult situations such as being in care or abusive families.

However, it was recognised that young people might need guidance about financial management. Related to independence is the relationship young people have with their parents. Several participants said that they feel guilty asking parents for money and that this would improve their relationships with them. A UBI for young people was also seen as a means of addressing some of the inequalities that exist in friendship groups, allowing poorer and richer children to socialise on an equal footing.

The negatives that participants associated with a UBI for people their age reflected those presented about the policy in general. Some were concerned that certain people may spend it frivolously on things that are not essential in life. This was also linked to a lack of financial literacy education.

Participants were asked to consider whether a UBI should be paid to parents or guardians, young people or split between the two. Paying directly to young people was seen as important in realising the potential benefits around independence, though this also amplified the concerns that the money would be spent ‘irresponsibly’. Paying wholly to parents was seen as problematic as some parents could choose to spend it on themselves or otherwise offer an additional means of control over their children. It was concluded that on balance that a split payment was preferred.

In the post-workshop survey, 15 preferred that 50% of the £40 payment be paid to the young person and 50% to their parent or guardian. Four preferred that 100% go to the parent or guardian and three to the young person.

## Discussion

Analysis by Parra-Mujica et al. (2023) and Villadsen et al. (2023) have provided quantitative evidence that young people’s mental health is affected by their socioeconomic background over which they have little or no control. The findings of this study provide rich, qualitative evidence on people’s lived experience that economic and welfare systems affect the wellbeing of young people, but, crucially, that young people also have a deep understanding of the pathways that these effects take. The focus on security, particularly when considering their futures, reflects existing evidence on the role of perceived insecurity. Kopasker, Montagna, and Bender (2018) use data from the British Household Panel Survey to assess the causal effect of various aspects of economic insecurity on mental health in the UK. They find that subjective economic insecurity – perceived future risks – are even more damaging to mental health than realised volatility. This reflects findings from a study of adults in ‘red wall’ constituencies in Wales and the North and Midlands of England, in which security of income was felt to be one of the most popular features of UBI (Johnson et al. 2023). In addition, there is good evidence that subjective social status as part of broader issues relating to inequality has an important role in determining health outcomes beyond objective measures (Johnson & Johnson 2019). Participants felt that UBI has the potential to address not only poverty, but also the mental (and physical) health harm affected by these subjective drivers.

Indeed, the data presented here support Murray and Webster’s (2022, 23), claim that young people believe that their ‘financial situation or relationship with work’ is putting ‘their health and wellbeing at risk’. This is a critical finding that elucidates social determinants and young people’s perception of them.

As such, this study provides important evidence from young people on the economic challenges they face at key life transition points, their preferences in relation to potential UBI schemes and the potential impact they believe they might have. A limitation of the study, however, is the female overrepresentation in the sample. This reflects existing, if developing, evidence on differences in male and female response rate to online surveys, both among students (Porter and Umbach 2006) and in the general population (Wu, Zhao, and Fils-Aime 2022). Efforts were made to recruit more young men and boys but the inability to obtain a more even gender split represents a challenge in giving a voice to participants with similar characteristics. Further research should be undertaken with male participants as well as in other parts of the country.

A limitation of the finding that young people do not prefer the most generous UBI scheme presented to them (as one would express if behaving as utility-maximising rational agents) is that people tend to choose the middle of three options, a phenomenon known in psychology as the theory of centrality preference (Shaw et al. 2000). The order of the schemes in this study was not randomised, as they formed a scale in terms of payment level and other orders could have resulted in confusion for respondents. Nonetheless, the finding suggests that some participants’ concerns about budgetary implications or the risks of poor spending choices did not result in substantial support for the lowest option. Indeed, in itself, Scheme 2 represents a major and radical welfare intervention.

## Conclusion

The participatory approach adopted presents rich qualitative evidence on underrepresented lived experience of the relationship between income and mental health. This complements quantitative evidence linking lower income and worse mental health outcomes in the UK (Parra-Mujica et al. 2023; Villadsen et al. 2023). Based on the pathways framework established by Johnson et al. (2022), UBI may mitigate this phenomenon by increasing incomes, especially among young people who are largely excluded from the present welfare system. To consider this, we conducted workshops with 28 young people in Bradford, UK to gather new data on young people’s views on the relationship between economic security and their mental health and of UBI.

Most participants in this study considered the current welfare system to be broadly unfair. There was also a large degree of consensus that it is failing young people, requiring them to make difficult trade-offs and sacrifices, with far-reaching impacts on their mental health. Most participants explicitly stated that a UBI would have some positive impacts on their mental health, and all participants registered their support for some form of UBI payment. Reasons suggested for the mental health benefits of UBI included a reduction in poverty, inequality and insecurity, but also extended to other non-economic determinants. These included a reduction in social stigma, more personal independence, enhanced relationships and an improved ability to plan for the future.

While there were differing perspectives on the level of UBI that participants would prefer to see implemented, the range of motivations for these different levels yielded a positive discussion. Participants showed the value and consideration that needs to be brought to questions of policy at this scale. There were indications from participants that they would like to see the retention of some conditional benefits in order to avoid people who currently receive them losing out under a flat-rate UBI system.

This article suggests that there is support among young people for a wider roll-out of UBI. Their support reflects an unprecedented vulnerability among young people to changes in employment and welfare. The data presented here suggest that policymakers need to regard engagement with young people as an essential means of achieving greater security and stability in society. More work is required, however, in order to understand perceptions of broader impacts of UBI on physical health, particularly among young people.
